# Root Canal Treatment of a Maxillary Second Molar with Two Palatal Canals: a Case Report

**Published:** 2015-12

**Authors:** MohammadReza Nabavizadeh, Abbas Abbaszadegan, Hosein Mirhadi, Yasmin Ghahramani

**Affiliations:** a Dept. of Endodontics, School of Dentistry, Shiraz University of Medical Sciences, Shiraz, Iran.

**Keywords:** Maxillary Second Molar, Two Palatal Canals, Root canal therapy

## Abstract

Careful understanding of internal anatomy of root canal system is crucial for successful endodontic treatment. The presence of two palatal canals in maxillary second molar is unusual but noteworthy as an aid to appropriate diagnosis and treatment. This paper reported a case of a maxillary right second molar with two root canals in the palatal root. The root canal treatment and case management were also explained.

## Introduction

A thorough knowledge of the root morphology and canal anatomy is mandatory to achieve the main objectives of root canal treatment, such as pain relief, root canal disinfection and prevention of reinfection. On the other hand, insufficient understanding of the canal morphology may lead to many of the challenges faced during root canal treatment. 

While it is common for maxillary second molars to have 3 roots with 3 canals, additional palatal canals are more common in maxillary first molars.[1-8] A single palatal root with two canals or two separate palatal roots have been described in these teeth in the literature. However, there were few clinical case reports exhibiting the presence of extra palatal canals and/or roots in maxillary second molars.[8-13] For instance, in the comprehensive studies performed by Shalabi, Green, and Vertucci, the presence of two palatal root canals in maxillary second molars has not been reported.[14-16]

Christi et al. reported 14 cases of maxillary second molar with two palatal roots over 40 years.[17] In 2012, Ghoddusi et al. reported a case of maxillary second molars that had two palatal canals.[18] A case of bilateral four-rooted maxillary second molar with two buccal and two palatal roots was reported by Alani in 2003.[19]

The aim of the present report was to describe nonsurgical endodontic treatment of a maxillary second molar with two palatal canals. 

## Case Report

A 52-year-old male patient with no history of systemic diseases was directed to the Department of Endodontics at Shiraz University of Medical Sciences for spontaneous pain in the maxillary right second molar. The pain steadily increased in intensity from mild to moderate, lasting for a few minutes.

**Figure 1:**
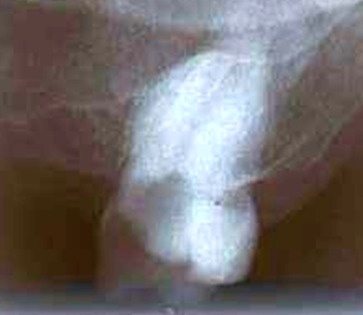
Initial radiograph of maxillary second molar

Examination showed that all teeth in the maxillary right posterior quadrant were extracted except the mentioned tooth. The patient did not indicate any history of trauma to the head region. Clinical and radiographic inspection of the maxillary right second molar revealed deep occlusal caries (Figure 1) with severe pain on probing by explorer.

**Figure 2:**
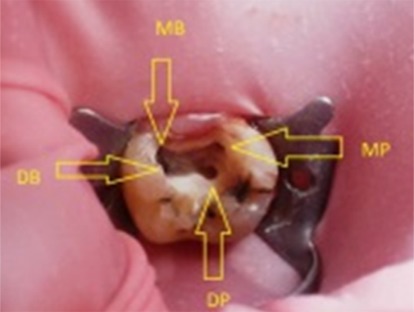
Access cavity with demonstrating arrows (MP: Mesiopalatal, DP: Distopalatal, MB: Mesiobuccal, DB: Distobuccal)

These findings and pulp vitality tests led to the diagnosis of irreversible pulpits, requiring root canal therapy. After anesthetizing the affected tooth and isolating it with a rubber dam, the standard access opening was prepared using a round diamond bur (No.4, MANI Inc.; Tochigi–Ken, Japan). Four canal orifices, two on each of the buccal and palatal canals, were confirmed by clinical evaluation of chamber floor. (Figure 2)

**Figure 3:**
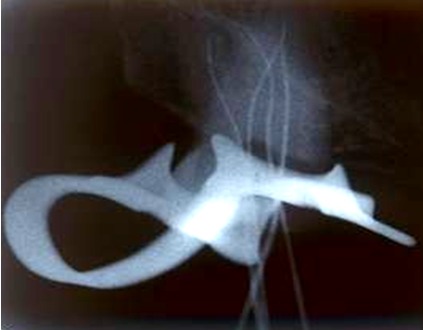
Working Length determination radiograph with four K-files.

An Apex locator (Root ZX; Morita, Tokyo, Japan) was used to estimate the working length of each canal. These lengths were confirmed by conventional intraoral periapical radiography. (Figure 3)

   

**Figure 4:**
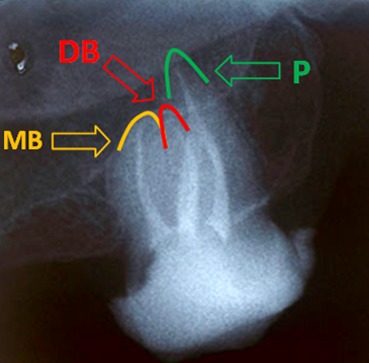
Final obturation. Yellow arrow shows the mesiobuccal apex, red and green arrows determine the distobuccal and palatal apices, respectively. Distopalatal and mesiopalatal canals merged into one apex.

NiTi rotary ProTapers and 5.25% NaOCl were employed to prepare and clean the root canals. Then, the canals were obturated by using cold lateral condensation technique with AH26 sealer (Dentsply DeTrey; Konstanz, Germany) and gutta-percha cones on the same visit. (Figure 4) After the temporary restoration of the access cavity with temporary filling material (Cavit; 3M, ESPE), the patient was referred for permanent restoration.

## Discussion

Peikoff et al.[20] reported six variations of a second maxillary molar in a retrospective study as Three distinct roots and canals (56%), three separate roots and four canals (two mesiobuccal canals) (22.7%), three roots and canals uniting mesiobuccal and distobuccal canals (9%), two separate roots and canals (6.9%), a single root with one canal (3.1%), and four roots or canals including two palatal canals (1.4 %).

According to this classification, our report falls into the latter group. The palatal root had two canals merging to one apex (type II Vertucci). Such cases have already been reported by Benenati[9] describing root canal therapy of an intact permanent maxillary second molar with two separate palatal canals and a palatogingival groove. Almeida et al.[11] reported a case of two palatal root canals in a maxillary second molar that was endodontically treated. Eskandarinezhad and Ghasemi[13] described non-surgical retreatment of maxillary second molar with two palatal root canals.

Palatal orifices in our report were located wider than that of the buccal orifices. This anatomic feature appears to be like the clinical photograph in the previous studies.[19, 21-22] Although radiographic evaluation is effective for locating anatomic variations, for the present case, the initial radiograph failed to detect two palatal canals.

Posterior location and superimposition of the anatomic structures on the radiographs are two important reasons for the possibility of failure to diagnose a second palatal root canal.

Owing to the challenges in interpreting the morphologic variations on radiographs, the use of operating microscope, visualization techniques, cone beam or spiral computed tomography scan are recommended. 

## Conclusion

The possibility of two palatal canals should be scrutinized in second maxillary molars. 
